# Biological Effects of Space Hypomagnetic Environment on Circadian Rhythm

**DOI:** 10.3389/fphys.2021.643943

**Published:** 2021-03-09

**Authors:** Xunwen Xue, Yasser F. Ali, Wanrong Luo, Caorui Liu, Guangming Zhou, Ning-Ang Liu

**Affiliations:** ^1^State Key Laboratory of Radiation Medicine and Protection, School of Radiation Medicine and Protection, Collaborative Innovation Center of Radiological Medicine of Jiangsu Higher Education Institutions, Soochow University, Suzhou, China; ^2^Academy of Space Life Sciences, Soochow University, Suzhou, China; ^3^Biophysics lab, Physics Department, Faculty of Science, Al-Azhar University, Nasr City, Egypt

**Keywords:** space hypomagnetic field, circadian rhythm, magnetoreception, chronobiology, cryptochrome

## Abstract

The intrinsic earth magnetic field (geomagnetic field, GMF) provides an essential environmental condition for most living organisms to adapt the solar cycle by rhythmically synchronizing physiological and behavioral processes. However, hypomagnetic field (HMF) of outer space, the Moon, and the Mars differs much from GMF, which poses a critical problem to astronauts during long-term interplanetary missions. Multiple experimental works have been devoted to the HMF effects on circadian rhythm and found that HMF perturbs circadian rhythms and profoundly contributes to health problems such as sleep disorders, altered metabolic as well as neurological diseases. By systemizing the latest progress on interdisciplinary cooperation between magnetobiology and chronobiology, this review sheds light on the health effects of HMF on circadian rhythms by elaborating the underlying circadian clock machinery and molecular processes.

## Introduction

A geomagnetic field (GMF) of about 50 microtesla (μT) is a vector field defined by both its intensity and direction at a given place. Earth’s magnetic poles are currently positioned near its geographical poles which make the compass usable for navigation. The conventional view is that the earth’s magnetic field is used as a backup to celestial (sun, polarization-based, or star) compasses. This view has originally arisen from an experiment done by Keeton (1969) when pigeons were held in a light-tight room with artificial lights 6 h out of phase with sun time, earlier or later. At release on sunny days at noon, bearing was 90° to the right or left of a home, depending on the direction of the clock shift. Under overcast conditions, however, the birds are accurately oriented; clearly, they are using a secondary compass. Applying a strong static magnetic field disrupts the homing on cloudy days, but not when the sun—the primary compass—is visible (Keeton, 1971). The GMF has protected our planet for millions of years and allowed species origination and evolution by effective protection from the solar stream of high-energy charged particles (Uffen, 1963; Wei et al., 2012; Tarduno et al., 2014).

There is no question that any kind of isolation from the GMF may interfere with biological processes and biochemical reactions. By the end of the 1960s, Soviet scientists made a significant contribution to the biological effects of a local GMF-shielded condition, so-called hypomagnetic field (HMF), during the space exploration missions (Zhadin, 2001). Changes in the enzyme activity of hydroxindolo-O-methyltransferase (HIOMT) which is responsible for melatonin biosynthesis in the pineal gland and retina and serotonin N-acetyltransferase (NAT) have been observed in response to variations in magnetic field strength (Cremer-Bartels et al., 1984). An independent study conducted by NASA on mice showed that the long-term lack of magnetic field greatly reduced the adaptability of the test animals (Wang et al., 2018). Additionally, leucopenia, low metabolic rate, increased mortality, and circadian rhythm disorders have been documented in association with absence of GMF.

The Earth’s rotation around its axis results in a molecular clock to which creatures have evolved to adapt their physiology with the solar cycle. In mammals, the circadian machinery is composed of two parts: a central pacemaker located in the hypothalamus, and local oscillators in the peripheral tissues such as liver, lung, kidney, and intestine. Peripheral clocks are considered to be synchronized by the master clock through means of synchronizing cues such as neuronal and hormonal signals to ensure coordinated physiological activity. Melatonin and insulin secretion, cardiovascular parameters, and blood pressure are examples of rhythmic functions that are monitored by the human circadian pacemaker. Epidemiologic studies indicate that circadian rhythm disruptions are associated with increased cancer risk (Sigurdardottir et al., 2012; Dickerman et al., 2016; Markt et al., 2016; Wendeu-Foyet and Menegaux, 2017). Thus, the circadian clock is critical to maintaining physiologic homeostasis and normal function of organisms.

The magnetic field may serve as a zeitgeber that is able to delay or advance the circadian rhythmicity. Brown (1960) found that the circadian rhythms of fiddler crabs and other organisms were affected by small changes in the intensity of GMF. Further studies have also shown that exposure to the HMF profoundly affected the central nervous system, resulting in structural change and functional alteration (Binhi and Sarimov, 2009). Ground-based research data of the negative effect of HMF on rhythmic functions, including neuroendocrinology, energy metabolism, oxidative stress (OS), and behavior, have been available to help study the risk uncertainty and development of efficient countermeasures.

## Parameters of HMF in Outer Space

Information on magnetic field classification and magnetic intensity in space can provide a scientific basis for simulating the exposure situation of low magnetic intensity on earth.

### Magnetic Field Classification

HMF is an extremely weak magnetic field with its total magnetic flux intensity of less than one-tenth of the GMF, while GMF is a geocentric axial dipole field with the greatest intensity at the magnetic poles and the smallest at the magnetic equator. The field intensity has a mean value of 50 μT (Thebault et al., 2015). The interstellar space magnetic field is much smaller than that of the Earth (Steinhilber et al., 2010). Zero magnetic field (Belyaev et al., 1997), near-zero (null) magnetic field (Bliss and Heppner, 1976; Rakosy-Tican et al., 2005), hypogeomagnetic field (Kopanev et al., 1979; Mo et al., 2011), low magnetic field (Negishi et al., 1999), low-level magnetic field (Martino and Castello, 2011), and extremely low magnetic field (Belyavskaya, 2001) are synonymous for describing the subgeomagnetic nature of the outer space. Mo et al. (2012b) proposed intensity values of “0 < |B| ≤ 5 μT” to lay the definition of HMF. The term “lunar magnetic field” has been adopted for a magnetic field strength that lies in the range of 0–300 nT, while “Mars magnetic field” has been released to describe range values of 300 nT to 5 μT.

### Magnetic Environment in Outer Space

In the past 50 years, the interplanetary magnetic field varies between 2 and 8 nT, with a mean value of 6.6 nT, corresponding to an increase of the solar magnetic flux (Steinhilber et al., 2010). The field strength of individual planets is very different from each other. For example, it is about 350–700 nT on Mercury, and the magnetic field at the Venus surface is much smaller than that at the Earth. At the surface of Jupiter and Saturn, it is more than a dozen times that of the Earth. The main destinations for human interplanetary exploration are the Moon and Mars. The Moon does not possess a global dipole magnetic field (Lin et al., 1998). Data obtained from several lunar orbiting spacecrafts indicate that the Moon’s minimal magnetic field intensity is usually several nanotesla and unevenly distributed (Shkuratov et al., 1999). The maximum field intensity is about 300 nT, mostly located in the highlands on the far side of the Moon (Lin et al., 1998; Berguig et al., 2013). “Magnetic refuges” are lunar regions of high residual magnetic field that can shield cosmic rays to some extent, where a human lunar base can be set up (Wieser et al., 2010) for lunar exploration projects. Mars is a multi-pole planet with many local magnetic fields where the southern hemisphere has a higher magnetic field. Cain et al. (2003) predicted that there might be some regions of high residual magnetic field at the surface of Mars with intensity up to several microtesla. These data indicate that the maximum value of interplanetary HMF intensity would not exceed 5 μT. The field strength of each planet is positively correlated with its mass, core radius, and rotational angular velocity. Solar wind activity can interfere with the planet’s magnetic field. As the core of the planet cools, its internal “magneto” slowly shuts down and fades away. In addition, celestial events such as asteroid strike may also induce the disappearance and uneven distribution of the magnetic field.

### Laboratory-Based Establishment of HMF

Biological laboratories developed acceptable methods for imitating the hypomagnetic environment of space in order to study the potential risk of exposure. (a) Compensation—large near-zero magnetic field working volumes can be obtained by using three sets of Helmholtz coils, oriented in the planes of the three natural dimensions and connected to three DC power supplies for compensating the three vectorial components of the Earth’s field (Zhang et al., 2004). Such an arrangement provides an interplanetary-like hypomagnetic working area (Agliassa et al., 2018). (b) Shielding—experimental volume completely surrounded by metal sheets made of high magnetic permeability alloy, such as Mu-metal, which deflects the force field by concentrating it within the metal substance. This method can successfully bring the field contained in the experimental zone down to about 0.2% of GMF or even lower (Zhang et al., 2017). Astatisation and superimposition of fields are two more methods that have been applied to reduce the ambient magnetic field or change the direction of the geomagnetic field vector by using the bar magnet.

## HMF Effect on Metabolism, Embryonic Development, and Neural Function

Mitochondria are the main sites for energy synthesis in mammals, in which ATP is synthesized by aerobic metabolism of glucose, fats, and amino acids. Mitochondria displayed considerable structural changes in mouse cardiomyocytes after staying at the hypomagnetic area with 10^5^ reduction of the geomagnetic field. Other complications, namely, focal lysis and thinning of myofibrils, focal degradation of the sarcoplasm, stimulation of phagocytosis, and marked reduction in the number of β-glycogen granules, have additionally been reported from disturbed biosynthesis of structural proteins under HMF (Nepomniashchikh et al., 1997). A recent *in vitro* study found that the glucose consumption and mitochondrial membrane potential of HMF-exposed skeletal muscle cells were significantly lower than those of the geomagnetic control (Fu et al., 2016); the level of intracellular genotoxic substance—reactive oxygen species (ROS)—was repressed in HMF-cultured human neuroblastoma cells (Zhang et al., 2017). In accordance with these findings, our recent study indicated that HMF simulated by geomagnetic shielding enhances radiation resistance by elevating the capacity of genomic stability maintenance in human bronchial epithelial cells (Xue et al., 2020). On the other hand, HMF has been reported to increase the lipid peroxide oxidation in the lung, liver, kidney, and small intestine of guinea pigs and mice after a short exposure in a shielded chamber (Babych, 1995, 1996). Changes in H_2_O_2_ production, active species (ROS), and energy consumption have been suggested as mechanisms by which HMF alters rhythmic metabolism (Zhang et al., 2017).

Iron is an important factor for maintaining cellular redox homeostasis, probably due to its chemical properties as it has unpaired electrons which make it capable of accepting or donating electrons. Iron can exist in two valence states, Fe(II) and Fe(III), whose magnetic properties are quite different. Ferrous iron (Fe^2+^) can be either paramagnetic with the effective spin 2 (high-spin state) or diamagnetic (low-spin state), while ferric iron (Fe^3+^) is all the time paramagnetic with an effective spin of 5/2 (high-spin state) or 1/2 (low-spin state) (Yang et al., 2018). All these states depend on the ligand atoms, and paramagnetic ions always interact with the magnetic field suggesting the involvement of magnetic field in iron metabolism in cells. Iron is generally present in iron–sulfur (Fe–S) cluster-containing proteins that participate in the control of gene expression, control of labile iron pool, and DNA damage recognition and repair (Brzóska et al., 2006). Here are few examples; an apo-protein of mammalian cytosolic aconitase, iron-regulatory protein 1 (IRP-1), binds to mRNA iron-responsive elements and consequently regulates the expression of several proteins involved in cellular iron metabolism. On the other hand, a number of DNA-binding proteins including DNA helicase XPD family members, DNA polymerases, and helicase–nuclease Dna2 were characterized as Fe–S proteins that play a major role in maintaining genome integrity (Golinelli-Cohen and Bouton, 2017).

GMF is required for normal development of organisms on the earth. Spinal curvature, malformed eyes, and retarded or blocked development were observed in larvae of a Japanese newt placed in a structure shielding a GMF by a factor of 10,000. An HMF exposure for 2 h could induce embryo malformation by altering the orientation of the mitotic spindle apparatus in *Xenopus* (Asashima et al., 1991; Mo et al., 2012a). For mouse primary embryos, HMF exposure increased the abortion rate of pregnant mice and disturbed the reorganization of the cytoskeleton as well as blastomere orientation (Osipenko et al., 2008; Fesenko et al., 2010). Magnetic shielding was reported to be associated with an increased incidence of somatic defects, bi-headedness, and intestinal protrusion (Wan et al., 2014). Decreased body weight and female fecundity were found in two species of rice planthoppers, *Laodelphax striatellus* and *Nilaparvata lugens*, that were grown in a zero-magnetic chamber for one month. Eventually, the mortality rate of HMF-exposed animals was significantly greater than that of the normal geomagnetic group (Kopanev et al., 1979).

HMF exposure has been shown to markedly decrease the work capacity, endurance, and behavioral activity of male Wistar rats (Levina et al., 1989). A previous study found that the content of γ-aminobutyric acid (GABA) in the cerebellum and basal ganglia of golden hamster decreased, while the content of taurine in the cerebellum gradually increased (Li et al., 2001). Alteration of these amino acid neurotransmitters was observed in patients with neurological diseases (Emir et al., 2012; Yuan et al., 2013; Menzie et al., 2014). Memory disorders have been noted in association with exposure to HMF. Impairment of long-term memory of taste avoidance was reported in chickens placed in reduced GMF structure of strength 700 nT (Wang et al., 2003). Consistently, HMF exposure was shown to lead to alteration in learning and memory ability of fruit flies (Zhang et al., 2004). Since the nervous system works via electrical signals, it should not be surprising that magnetic field may interfere with brain functions such as memory mechanisms.

## Association Between HMF Exposure and the Secretion of Melatonin and Norepinephrine, Two Markers of the Circadian System

Endogenous biological clocks help living organisms adapt to the Earth’s 24 h cycle. These intrinsic oscillators are autonomous systems for the maintenance of biological rhythms even in the absence of external time cues. However, circadian rhythms can be reset or entrained by environmental cues such as light, temperature, and lifestyle. Small changes in the intensity of the earth’s magnetic field have been found to affect profoundly the daily activity pattern of different species (Bliss and Heppner, 1976; Zamoshchina et al., 2012; Mo et al., 2015). Melatonin is a pineal gland hormone which functions as an internal synchronizer to adequately time the organism’s physiology in the daily and seasonal demands. Darkness promotes melatonin synthesis and release; therefore, its concentration falls during daylight. Besides the well-known function of melatonin in circadian rhythm control, melatonin and its derivatives have a free radical scavenger and antioxidant effect. By modulating cytokine and OS levels, melatonin has also been reported to play dual roles in immune modulation (Carrillo-Vico et al., 2013). Changes in GMF magnitude have been found to disturb the 24 h circadian melatonin secretion rhythm (Jia et al., 2014), resulting in negative consequences in decreasing the body’s antioxidant capacity. The effect of magnetic fields on pineal melatonin may require retinal stimulation by light. Optic nerve transections or complete darkness blocks the ability of magnetic field exposure to suppress pineal gland N-acetyl transferase activity and melatonin content. Hence, light activation of photoreceptors is essential for the magnetosensitivity (Reuss and Olcese, 1986). On the other hand, misalignment between endogenous clock and activity pattern of mice has been early reported after staying at an HMF structure (Zamoshchina et al., 2012).

Plasma norepinephrine (NE) released from sympathetic nerves and adrenal medulla kept under light–dark or constant dark conditions displays significant daily variation. Rhythmic variation of NE in the perivascular space of the pineal gland is a primary input for synchronizing pineal melatonin synthesis and clock gene transcription. The rhythmic oscillations of clock genes *BMAL1*, *PER2*, *CRY2*, *NR1D1*, *Dbp*, and a key enzyme of melatonin synthesis, arylalkylamine-N-acetyltransferase (*Aanat*) in cultured pinealocytes, are synchronized by NE stimulation (Andrade-Silva et al., 2014). Noradrenergic activities in the brainstem of golden hamsters were shown to be affected as both the content of noradrenaline and the density of noradrenaline immunopositive neurons in the tissue decreased significantly after a long stay in a near-zero magnetic environment (Zhang et al., 2007).

Furthermore, some experiments have revealed that the circadian activity of house sparrow has been entrained by a cycle of change in the intensity of the vertical component of the Earth’s MF (Bliss and Heppner, 1976) suggesting that diurnal geomagnetic variation could be a secondary zeitgeber for biological circadian rhythms in addition to the primary night–day light cycle. Consistently, decrease in general activity and disorder in circadian drinking rhythm accompanied with an increase in thermal hyperalgesia were recorded in laboratory rodents in response to 30 days in an HMF shelter (Mo et al., 2015).

## Cryptochromes Mediating the Light-Dependent Magnetic Sensitivity

In the course of evolution, cryptochromes (CRYs) are the descendants of DNA photolyases which can be classified into three groups: plant CRY, animal CRY, and CRY-DASH proteins (Cashmore et al., 1999; Chaves et al., 2011; Haug et al., 2015; Kutta et al., 2017; Ozturk, 2017). CRY proteins have long been known to function as circadian photoreceptors and to participate in the circadian feedback loop, mediating the light resetting of the 24 h clock located in the nuclei of nerve and certain retinal cells.

CRY4 currently seems to be the only bird CRY that binds FAD at physiological conditions, which is a definitive requirement for the ability to function as light-dependent magnetoreceptors for seasonal migration (Watari et al., 2012; Mitsui et al., 2015; Günther et al., 2018; Pinzon-Rodriguez et al., 2018). However, the light-dependent magnetosensing function of avian CRYs has not been yet identified in their mammalian counterparts.

**FIGURE 1:**
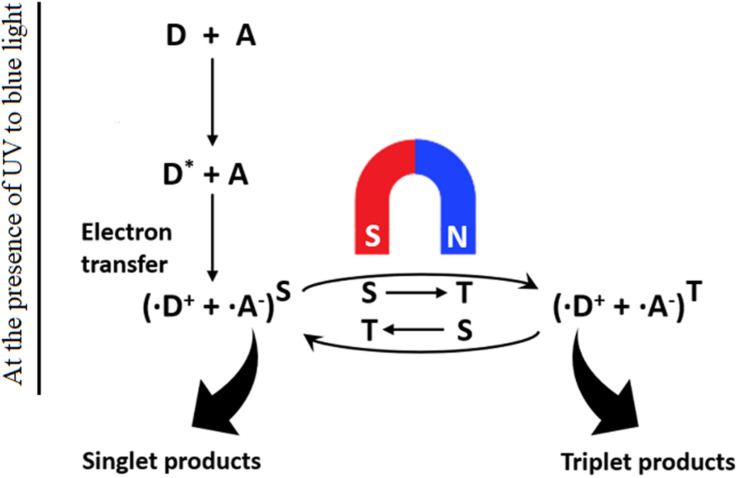
Schematics of the light-dependent chemical magnetoreception model. CRY proteins (D) exist in an inactive ground state. Absorption of UV or blue light promotes electron transfer to an acceptor atom (A), resulting in a radical pair (D^+^ and A^–^) that further undergoes magnetically singlet–triplet interconversion. (Modified from Ritz et al., 2000).

Two main models of magnetic field biosensing have gained scientific consensus. The first is a magnetite-based force transduction model. Mechanically, magnetite crystals connected to the internal surface of the cell cytomembrane through unstretched cytoskeletal filaments provide a biochemical mechanism for force transduction. The crystalline magnetite nanoparticles transduce GMF information to the nervous system through secondary receptors, such as stretch receptors or mechanoreceptors. Examples of this model include magnetosensitive retina in the eyes of some birds and magnetite chains in the nasal cavity of salmonid fish (Wiltschko and Wiltschko, 2005; Binhi and Prato, 2017). The second modality is called radical-pair model “chemical compass” (Figure 1). In this model, the retina absorbs light energy to activate the photosensitive protein CRY to mediate spin-coupled radical pair formation, which provides a chemical magnetosensing platform in a variety of organisms (Ritz et al., 2010; Dodson et al., 2013; Zoltowski et al., 2019). The photoactivation-generated radical pairs can exist in either singlet (↑↓) or triplet (↑↑) spin state in the presence of the applied magnetic field. The electrons from singlet or triplet radical pairs can undergo a spin-selective reaction to produce the singlet or triplet product. In this way, the environmental magnetic field is reflected and sensed by the different ratio of singlet/triplet products in organisms, which results in either different reaction kinetics or product composition for the chemical reaction. Such photoexcitation-based immediate response would provide a means for the organisms to detect even very weak magnetic fields (Yoshii et al., 2009; Liedvogel and Mouritsen, 2010).

To explore the mechanism how photoenzyme CRY was involved in the cellular magnetoreception-modulating process, a combination of genomic techniques, microscopy, and protein structural modeling was applied to identify a magnetoreceptor protein MagR, which acts like a compass needle to bind with iron and forms a rod-shaped complex when physically associated with CRY in the head of Drosophila (Qin et al., 2016). This Cry/MagR magnetosensor complex composed of MagR polymerized into a linear protein complex and was wrapped helically by CRYs. Such nanostructure offers the complex the light-dependent property and an intrinsic magnetic moment that rotates to align itself with geomagnetic field lines.

CRY has been shown to participate in a molecular complex feedback loop to generate the circadian oscillation in response to magnetic fields. CRY-mutant fruit flies lost their ability to sense GMF for orientation and navigation. Moreover, by using the transgenic approach, reintroduction of human CRY was proved to rescue the magnetoreception pathway in CRY loss-of-function mutated drosophila (Yoshii et al., 2009). Wild-type flies have shown obvious natural response to the magnetic field under full-spectrum light, while CRY-deficient flies expressed neither naive nor trained responses to the magnetic field under the same light condition. Further study indicated that the blue-light part of the spectrum (<420 nm) may play an important role in CRY-mediated behavior in response to magnetic field (Gegear et al., 2008). These data indicate that CRY may serve as a magnetic compass detector as well as a mediator of circadian rhythms.

## Conclusion and Perspectives

On earth or traveling to the International Space Station, people are protected from much of cosmic radiation by the Earth’s magnetosphere. However, astronauts on longer flights beyond the low orbit of the earth where the geomagnetic field is negligible in strength are at greater risk. Indeed, innovative designs that form large electromagnetic fields around the craft or base, usually through the superconducting solenoids, has been suggested in order to mimic the protection of the Earth’s magnetosphere. In addition, ground-based studies on interplanetary-like HMF have also revealed changes in structure and function of the central nervous system, embryonic development, hormone-release synchronization, and circadian rhythm in animal and cells. Inconsistency and seemingly contradictory observations are shown in literatures that may arise from difference in parameters of magnetic field and characteristics of the study subject. This implies the need for additional research on a higher number of subjects to better understand health issues during travel beyond the Earth’s orbit and into deep space.

## Author Contributions

XX and YA drafted the manuscript. N-AL and GZ developed the final version. WL and CL contributed to writing and to critical review the manuscript. All authors approved the final version.

## Conflict of Interest

The authors declare that the research was conducted in the absence of any commercial or financial relationships that could be construed as a potential conflict of interest.
